# The Hippocampal Engram as a Memory Index

**DOI:** 10.1177/1179069518815942

**Published:** 2018-12-02

**Authors:** Kazumasa Z Tanaka, Thomas J McHugh

**Affiliations:** 1 Laboratory for Circuit and Behavioral Physiology, RIKEN Center for Brain Science, Wako, Japan; 2 Department of Life Sciences, Graduate School of Arts and Sciences, The University of Tokyo, Tokyo, Japan

**Keywords:** Hippocampus, engram, memory, plasticity, place cell

## Abstract

The hippocampus encodes memories for past events, but the nature of the hippocampal code subserving this function remains unclear. A prevailing idea, strongly supported by hippocampal physiology, is the Cognitive Map Theory. In this view, episodic memories are anchored to spatial domains, or allocentric frameworks, of experiences, with the hippocampus providing a stable representation of external space. On the other hand, recent studies using Immediate Early Genes (IEGs) as a proxy of neuronal activation support the Memory Index Theory. This idea posits that the hippocampal memory trace serves as an index for a cortical representation of memory (a map for internal representation) and hypothesizes the primary hippocampal function is to reinstate the pattern of cortical activity present during encoding. Our recent findings provide a unitary view on these two fundamentally different theories. In the hippocampal CA1 region the activity of c-Fos expressing pyramidal neurons reliably reflects the identity of the context the animal is experiencing in an index-like fashion, while spikes from other active pyramidal cells provide spatial information that is stable over a long period of time. These two distinct ensembles of hippocampal neurons suggest heterogeneous roles for subsets of hippocampus neurons in memory.

**Comment on:** Tanaka KZ, He H, Tomar A, Niisato K, Huang AJY, McHugh TJ. The hippocampal engram maps experience but not place. *Science*. 2018;361:392–397. doi:10.1126/science.aat5397. https://www.ncbi.nlm.nih.gov/pubmed/30049878

Multiple lines of evidence suggest that the hippocampus is indispensable for the encoding and storage of episodic memories. Human patients with damage to the medial temporal lobe exhibit profound anterograde amnesia, specifically for the autobiographical memories that reflect our experiences.^1,2^ Studies in animal models are consistent with these observations, with lesions or more specific manipulations, such as pharmacological or genetic interventions that impair hippocampal function, revealing a pattern of stark contextual and episodic memory deficits.^3^ For example, suppression of hippocampal activity impairs spatial or contextual learning, while the same manipulation does not affect simple tone–shock association.^4,5^ Thus, a key question that can be explored in model animals is how does neuronal activity in the hippocampus supports these types of memories.

A long-suggested mechanism of memory storage is activity-dependent synaptic plasticity. Previous work has indeed shown that long-lasting changes in synaptic efficacy are a consequence of hippocampal learning.^6^ This experimental evidence supports the hypotheses of Morris and others that long-lasting synaptic changes are not merely an outcome of neuronal activity, but rather serve as physical traces of a memory in a neuronal network.^7,8^ When animals retrieve memory, this memory trace would help reproduce a pattern of activity, even from a partial input, that represents a specific aspect of original experience—a network manifestation of the influential idea postulated by Donald Hebb’s “fire together, wire together” model.^9^

In a freely behaving animal, the activity of hippocampal neurons is modulated by the animal’s current location in space, hence the moniker “place cells.”^10^ In any given environment, a subset of hippocampal neurons are recruited and each represents a specific location in space. Thus, collectively, the active ensemble represents a spatial map of the animal’s current experience (Figure 1A). Based on his discovery of these place cells, O’Keefe and Nadel^11^ expanded their interpretation of this spatial map and proposed that activity of place cells in the hippocampus provides locale information to define an allocentric framework for a given cognitive domain on which objects and events can be related (the Cognitive Map Theory). An implication of this theory is that if memory is anchored to the spatial representations, then for memories to be stable and long-lasting, the underlying map of space should also be stable and reproducible. Early work to test this hypothesis was largely supportive, finding even when an animal returns to a maze 6 months after the initial training, the hippocampal neurons are able to respond in the same location.^12^ Moreover, receptors and signaling molecules known to be crucial for plasticity have been found to contribute to the stabilization of spatial map^13^; but also see Jeffery and Hayman.^14^ For example, pharmacological blockade of N-methyl-D-aspartate (NMDA) receptors in the hippocampus impairs stable formation of place fields.^15^ Together, these findings support the idea that the hippocampus encodes spatial memory and that synaptic plasticity modifies connections within the hippocampal network so that it can regenerate the same pattern of activity—firing at specific locations—even long after encoding.

**Figure 1.**
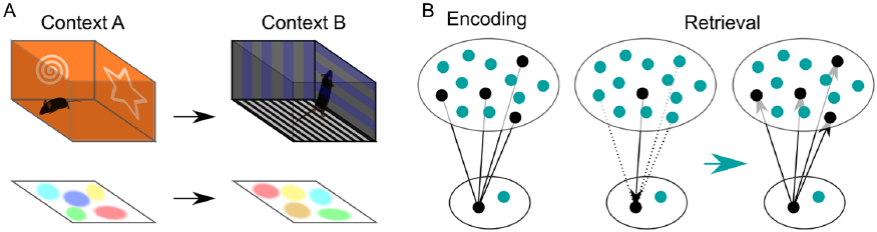
Two theories addressing a primary role of the hippocampus for episodic memory. (A) A subset of pyramidal neurons in the hippocampus is recruited for a given context and represents a specific location within the environment. O’Keefe and Nadel proposed spatial maps in the hippocampus being substrates for episodic memories. (B) Memory Index Theory assumes memory trace being a strengthened link between hippocampal and cortical representations. Once this memory trace is established during encoding, a partial input is sufficient to reactivate the complete memory representation in the hippocampus. This reinstates the original pattern of activity in the cortex and produces a sense of memory recall.

There is a second line of experimental evidence that further strengthens the links between plasticity and memory in the hippocampus. In these studies, the increased transcription or translation of immediate early genes (IEGs)^16,17^ is used as a proxy of neuronal activity, as their induction reflects patterned neuronal activity associated with synaptic plasticity. In an influential study, Guzowski and colleagues reported that hippocampal neurons reactivate transcription of IEGs when animals explore the same environment twice, but distinct ensembles are engaged when they experience two different environments.^18^ Importantly, IEG reactivation was observed even when animals were in a different behavioral state. Using a transgenic approach to explore this same phenomena, Tayler and colleagues employed the c-Fos-tTA mice (TetTag mice), which permits the labeling of active neurons during memory encoding via the presence (Dox ON/System OFF) or absence (Dox OFF/System ON) of the antibiotic doxycycline in the food. They observed significant IEG reactivation in the hippocampus and related cortical areas during freezing behavior following contextual fear conditioning.^19^ This finding suggests that IEG reactivation is associated with the retrieval of contextual memory rather than resampling of the same spatial locations, as a freezing mouse is not actively exploring the environment. Recent studies combining TetTag mice and genetic intervention of neuronal activity provided an even stronger link between ensembles of neurons and memory. Using optogenetics, Liu and colleagues artificially activated a subset of granule cells in the dentate gyrus that underwent IEG activation during contextual fear conditioning.^20^ This manipulation produced freezing, the behavioral correlate of memory recall in the task, even when the animal was in safe context, suggesting that activity of these neurons can trigger memory recall. Interestingly, when the activity of these neurons was suppressed during a memory test in the originally conditioned context, not only did the animals show reduced freezing behavior but also IEG reactivation of cortical neurons was compromised, suggesting the activity of the memory-bearing cells in the hippocampus is crucial both for behavior and for the activation of the memory in downstream regions.^21^ While these results are hard to interpret in the framework of place cells/Cognitive Map Theory, they are very congruent with the Memory Index Theory of Teyler and DiScenna.^22^ Their 1986 paper, building on the Simple Memory Theory of David Marr^23^ as well as others, postulated a unique role of the hippocampus in episodic memory, suggesting that the hippocampus contains an index to provide rapid and efficient access to memory content stored in neocortical areas. During memory encoding, hippocampal synapses responding to neocortical inputs are strengthened, such that a partial input later fed into this structure is able to recreate the original pattern of the activity in the neocortex (Figure 1B). In essence, this theory dissociates two fundamental aspects of memory device—reproduction of activity and representation of the episode—and assigns the former as the primary role of the hippocampus. In other words, the hippocampus stores memory of the co-occurring neocortical activity pattern.

Although the Cognitive Map Theory and Memory Index Theory are not mutually exclusive, it still remained unclear on how to reconcile them. While it has been long assumed that IEG expression in the hippocampus captures activity of place cells,^24^ the literature is riddle with contradictions. First, the location-specific firing of place cells is not sufficient to induce IEG expression in the hippocampus.^25,26^ This suggests that IEG expression may capture a specific pattern of activity, rather than simply reflect the increased spiking activity of place cells. Second, IEG expression in the hippocampus does not require physical exploration of an environment, more strongly suggesting dissociation of place cell firing from IEG induction.^19^ These reports make it difficult to link place cell physiology and contextual representation defined by IEG expression.

To address this question and provide novel insights on the hippocampal coding of memory, we conducted tetrode recording from CA1 pyramidal cells in freely moving c-Fos-tTA mice.^27^ To physiologically identify c-Fos positive cells, we injected AAV-TRE-ChR2-EYFP into the hippocampus to express light-responsive ChR2 in the positive neurons and allow their optogenetic identification. In the experiment, we recorded 3 sessions. First, animals explored a novel chamber (Context A, encoding) in the Dox OFF condition, allowing capture of the memory engram. The subset of CA1 pyramidal neurons that expressed c-Fos in this session was labeled with ChR2 and later identified through light-responsive spikes. The next day, after being returned to the Dox ON condition, the animals again explored the same chamber (Context A, recall) to assess neuronal activity during contextual memory recall. Finally, they were allowed to explore a novel context B, to examine context-specific activity.

As expected, the exploration of the novel context triggered c-Fos expression in a subset of CA1 pyramidal neurons, and we found that most of these neurons had place fields in the encoding environment. However, many other CA1 cells, not expressing c-Fos, also demonstrated location-specific firing in the chamber. Thus, only a fraction of place cells, ~1/4-1/3 of the active population, undergo c-Fos induction during contextual encoding. Given the use of c-Fos expression as a marker of neuronal activity, these data raise the question: is this special population of place cells more active than others? The answer is both yes and no. On average, these c-Fos-positive cells had higher mean firing rates (more spikes), but their peak firing rates are comparable to the negative population. Consequently, their place fields were slightly larger and their spikes carried lower spatial information. Therefore, c-Fos-positive CA1 neurons are place cells, slightly less spatially tuned, and while we can assume c-Fos expression is a readout of neuronal activity, we cannot conclude that a lack of c-Fos expression indicates no neuronal activity.

Then what is the pattern of activity that c-Fos induction captures? We noticed that c-Fos-positive place cells demonstrated an increased number of spike bursts compared to the other place cells during contextual encoding. Interestingly, these bursts of spikes were strongly paced at 6-12 Hz, the theta frequency that dominates the population activity of the hippocampus.^28^ These theta burst events (repetitive bursts paced at 6-12 Hz) were more frequent and longer in c-Fos-positive place cells. In brain slice, this exact pattern of activity can induce a potent form of long-term potentiation.^29^ Thus, our finding strongly suggests that these theta bursts, rather than simply higher activity, is the trigger for c-Fos expression. Moreover, we found spikes from c-Fos-positive place cells are more entrained by fast gamma oscillations in the hippocampal local field potential, suggesting an involvement of entorhinal inputs in this burst induction.^30^

Given that hippocampal CA1 place cells are a mixed population of c-Fos-positive and negative neurons, how can we reconcile the Cognitive Map Theory and the Memory Index Theory? One prediction in line with the Cognitive Map Theory is that c-Fos-positive place cells comprise the stable spatial map to which the memory is anchored. To test this prediction, we examined location-specific firing of these 2 types of place cells when animals returned to the same context the next morning. Contrary to these expectations, we found that c-Fos-positive place cells were preferentially spatially unstable, typically remapping their firing locations, while c-Fos-negative place cells showed high spatial stability between the 2 visits (Figure 2A). This observation indicates that the neuronal ensemble containing the stable spatial and navigational information is composed not of the engram neurons, but rather the c-Fos-negative place cells. Then what is represented in activity of engram cells and how do they support memory? Interestingly, these 2 types of place cells responded quite differently to the second, non-encoding context B. When animals explored context B, c-Fos-positive place cells dramatically change their firing rates, with many becoming virtually silent, in contrast to the c-Fos-negative place cells, which often shifted their firing locations but with comparable firing rates. Importantly, these firing rates that highly correlate with contextual identity (Figure 2B), but not with specific locations, emerged as soon as the animals were placed in the context, suggesting that the rapid reactivation of contextual representation by engram neurons can drive contextual memory recall.

**Figure 2.**
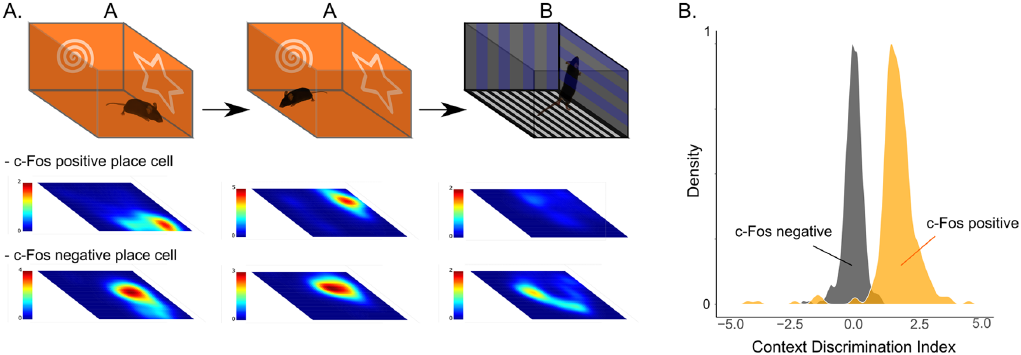
Two distinct memory traces in the hippocampal CA1. (A) Heat maps showing firing locations of representative c-Fos-positive/negative place cells when animal is exposed to a novel context A (left), a familiar context A (middle), and a novel context B (right). (B) Context Discrimination Index defined from firing rate correlations of c-Fos-positive (orange) or negative (gray) place cells when animal explores context A/A or A/B.

Our study revealed 2 types of memory traces allocated to distinct ensembles of neurons in the hippocampus. One encodes a map of the animal’s environment and stably exhibits location-specific firing. The second set of neurons has unstable spatial information between visits, but their net activity stably and rapidly reflects contextual identity. This neuronal ensemble (the so-called memory engram) is a stronger candidate for a neuronal substrate of the memory index, as their context-specific activity may efficiently reproduce the pattern of cortical activity present during the encoding of a specific episode. Although these 2 memory traces would conjunctively support distinct domains of episodic memory, as manipulation of engram results in global remapping of the stable place cell population,^31^ the Memory Index Theory predicts that their primary roles are fundamentally different and thus their activity would have distinct impact on the physiology of downstream areas. Future studies are needed to elucidate how these 2 types of memory traces interact with the extra-hippocampal circuits and support episodic memory.

One further implication of these data is that during memory encoding, these 2 neuronal ensembles may undergo heterogeneous synaptic plasticity events. Previous work suggests that the synaptic plasticity underlying the formation of place fields in CA1 requires conjunctive inputs both from CA3 and the entorhinal cortex.^32,33^ This process depends on activation of NMDA receptors and presumably occurs both in c-Fos-positive and negative place cells. Our in vivo data demonstrate that c-Fos-positive cells uniquely exhibit theta-paced bursts when the animal repeatedly entered the place field. An abundant amount of in vitro physiology suggests that these subsequent burst events can lead to a form of long-term potentiation that requires brain-derived neurotrophic factor (BDNF) secretion.^29^ These heterogeneous alterations of neuronal physiology are likely to underlie formation of the distinct memory representations in the hippocampus.

Another significant question is why engram neurons remap in the same context. One hypothesis is that this supports the temporal domain of contextual memories. Animals can discriminate temporal components of contexts, thus shifts of firing locations might help disentangling 2 temporally remote events in the physical place. Another possibility is that this remapping subserves strengthening the spatial map represented by the more stable c-Fos-negative place cells. It is thought that coactivity of hippocampal neurons may consolidate previously formed spatial maps.^34^ Shifting spatial firing locations of c-Fos-positive neurons would increase the number of sequences they could participate in, enhancing the consolidation of the other spatially stable cells. Although these 2 possibilities are not mutually exclusive, tests of these ideas would deepen our understanding on what this type of plasticity provides to the neuronal networks supporting memory.
